# Supplementary material for: Combining explainable machine learning, demographic and multi-omic data to inform precision medicine strategies for inflammatory bowel disease
Source: PLoS One. 2022 Feb 23;17(2):e0263248. doi: 10.1371/journal.pone.0263248 (PMC8865677; doi:10.1371/journal.pone.0263248)
Supplement: S1 Fig — Here we show box plots (left) of mean absolute error values (as percentages) computed during 10-fold cross validation. The horizontal line in each boxplot is the median of the MAE over 10 folds, where each of the test folds has ~3 randomly chosen patients. Note all target drug responses have been normalized on a scale of 0–1 and here we show percentages of MAE values. On the right we report median, average and standard deviation MAE as percentages for each ML method. We computed the predictive error using different combination of demographic features; (a) age, gender, condition, resection area and smoking, or (b) age, gender, condition, resection area or (c) age, gender, condition only. (DOCX) [file pone.0263248.s002.docx]

**Figure S1. Comparison of ML model error rates for the prediction of BIRB796 (at 10nM) drug response for different combinations of demographic features.**
