# Supplementary material for: Combining explainable machine learning, demographic and multi-omic data to inform precision medicine strategies for inflammatory bowel disease
Source: PLoS One. 2022 Feb 23;17(2):e0263248. doi: 10.1371/journal.pone.0263248 (PMC8865677; doi:10.1371/journal.pone.0263248)
Supplement: S2 Fig — (DOCX) [file pone.0263248.s003.docx]

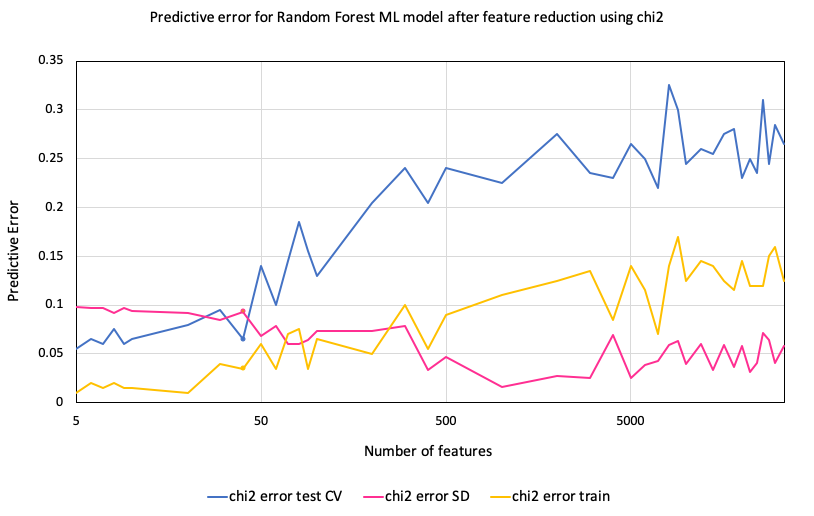


**Figure S2. Results of Chi2 test to sequentially remove SNP (genomic) features and observe effect on model MAE rate.**
