# Supplementary material for: Combining explainable machine learning, demographic and multi-omic data to inform precision medicine strategies for inflammatory bowel disease
Source: PLoS One. 2022 Feb 23;17(2):e0263248. doi: 10.1371/journal.pone.0263248 (PMC8865677; doi:10.1371/journal.pone.0263248)
Supplement: S3 Fig — RNA-seq data available for 14 of the 25 patients. Shown here are the top ranked SNPs from model explanation of best “demographic+medicinal+SNPs” model, if those SNP alleles showed correlation with gene expression (TMM values for corresponding genes) or if they showed correlation with transcript lengths of respective patients using Spearman’s Rho (rs) (> 0.3). Box plots compare the SNP alleles versus their distributions of either TMM values or transcript lengths (bp) for the gene of origin of the SNP. Each box plot is labelled (title) with the “gene of origin of SNP”-“rank of SNP in Table 2” (“if correlated with TMM or Length”). The x-axis denoted the SNP alleles featured in the patient population that are being compared; “Ref” denotes reference allele and “Het” denotes a heterozygous SNP. (DOCX) [file pone.0263248.s004.docx]

**Figure S3. Validation of 19 most impactful genomic SNP features to drug response prediction using RNA-seq data.**
