# Supplementary material for: Combining explainable machine learning, demographic and multi-omic data to inform precision medicine strategies for inflammatory bowel disease
Source: PLoS One. 2022 Feb 23;17(2):e0263248. doi: 10.1371/journal.pone.0263248 (PMC8865677; doi:10.1371/journal.pone.0263248)
Supplement: S1 Table — As identified from target validation.org. Those SNPs selected (one per gene) as non-synonymous or else the most correlated with RNA-seq data are highlighted in bold. (DOCX) [file pone.0263248.s005.docx]

**Table S1. Top 10 known genes associated with Ulcerative colitis and Crohn’s disease.**

| Gene | Location on GRCh37 | SNPs within this region |
| --- | --- | --- |
| JAK2 | Chromosome 9: 4,984,390-5,129,948 forward strand. | chr9_5090641; chr9_5090934; **chr9_5050706** |
| NOD2 | Chromosome 16: 50,727,499-50,767,952 forward strand. | chr16_50733859; chr16_50731096; chr16_50733374**; chr16_50744624**; chr16_50745199; chr16_50745583; chr16_50745655 |
| JAK3 | Chromosome 19:  [17,935,589-17,958,880](http://grch37.ensembl.org/Homo_sapiens/Location/View?r=19:17935589-17958880)  reverse strand. | **chr19_17937429**; chr19_17937758; chr19_17941294; chr19_17948732; chr19_17951178; chr19_17952185 |
| TET2 | Chromosome 4:  106,067,032-106,200,973 forward strand. | **chr4_106196829**; chr4_106196951 |
| GABRA2 | Chromosome 4: [46,245,565-46,477,247](http://grch37.ensembl.org/Homo_sapiens/Location/View?r=4:46245565-46477247) reverse strand. | **chr4_46334702** |
| IL12B | Chromosome 5:  158,741,788-158,757,495 reverse strand. | **chr5_158750013** |
| TYK2 | Chromosome 19: 10,461,205-10,491,248 reverse strand. | chr19_10468668; chr19_10473138; **chr19_10475652**; chr19_10475760; chr19_10477067; chr19_10478945 |
| PRDM1 | Chromosome 6: 106,441,338-106,557,814 forward strand. | chr6_106534419; **chr6_106547372;** chr6_106555025 |
| SMAD3 | Chromosome 15:  67,356,101-67,487,507 forward strand. | - |
| PRKCB | Chromosome 16: 23,847,304-24,231,932 forward strand. | chr16_24202458; chr16_23848762; chr16_24135112; **chr16_24166130** |
| IL10 | Chromosome 1:  206,940,947-206,947,886 reverse strand. | - |
| STAT3 | Chromosome 17: 40,465,342-40,540,500 reverse strand. | - |
| ITGA4 | Chromosome 2: 182,321,929-182,403,667 forward strand. | **chr2_182347072**; chr2_182392143  chr2_182399097; chr2_182399685 |
| TNF | Chromosome 6:  31,543,342-31,546,113 forward strand. | - |
| IL2RA | Chromosome 10:  6,052,652-6,104,333 reverse strand. | chr10_6066195; **chr10_6066200** |
| MAPK14 | Chromosome 6: 35,995,454-36,079,013 forward strand | - |
