# Supplementary material for: Combining explainable machine learning, demographic and multi-omic data to inform precision medicine strategies for inflammatory bowel disease
Source: PLoS One. 2022 Feb 23;17(2):e0263248. doi: 10.1371/journal.pone.0263248 (PMC8865677; doi:10.1371/journal.pone.0263248)
Supplement: S2 Table — (DOCX) [file pone.0263248.s006.docx]

**Table S2. Features specific to the model explanation (among the top 20 most impactful features) of a single tested drug or dosage from the 5 compared in this study.**

| BIRB796 10nM | BIRB796 100nM | SEBPRED 1uM | SEBPRED 100nM | 5ASA |
| --- | --- | --- | --- | --- |
| chr16_50733374 | chr10_6066200 | chr19_17952185 | chr16_24202458 | chr19_10475760 |
| chr16_50731096 |  | Adalimumab | chr4_106196951 | Nocurrentmeds |
| chr10_102891680 |  |  | chr16_24135112 | chr4_46334702 |
| chr7_87810955 |  |  |  | chr16_23848762 |
| chr19_7747445 |  |  |  | chr19_10468668 |
| chr5_178409927 |  |  |  |  |
