# Supplementary material for: Combining explainable machine learning, demographic and multi-omic data to inform precision medicine strategies for inflammatory bowel disease
Source: PLoS One. 2022 Feb 23;17(2):e0263248. doi: 10.1371/journal.pone.0263248 (PMC8865677; doi:10.1371/journal.pone.0263248)
Supplement: S3 Table — Pre and post alignment. (DOCX) [file pone.0263248.s007.docx]

**Table S3. Mapping statistics for the 14 RNA-seq samples. Pre and post alignment.**

| Sample | Read Number | Number Aligned Reads | % All Reads Aligned | Number Uniquely Aligned Reads | % All Reads Uniquely Aligned | % All Reads Duplicates | Number Uniquely Aligned Reads Post Remove Duplicates | % All Reads Uniquely Aligned Post Remove Duplicates |
| --- | --- | --- | --- | --- | --- | --- | --- | --- |
| P15_061 | 104,646,050 | 94,044,634 | 89.9 | 78,543,844 | 75.1 | 31.4 | 45,697,354 | 43.7 |
| P15_058 | 115,555,286 | 106,787,708 | 92.4 | 88,515,674 | 76.6 | 36.7 | 46,071,758 | 39.9 |
| P16_005 | 98,311,168 | 88,322,668 | 89.8 | 72,433,176 | 73.7 | 47.0 | 26,262,960 | 26.7 |
| P15_067 | 99,053,628 | 88,977,752 | 89.8 | 73,808,804 | 74.5 | 34.4 | 39,776,424 | 40.2 |
| P15_030 | 136,515,692 | 129,521,170 | 94.9 | 111,184,006 | 81.4 | 40.0 | 56,565,480 | 41.4 |
| P15_002 | 90,558,340 | 85,526,626 | 94.4 | 70,513,954 | 77.9 | 38.6 | 35,533,224 | 39.2 |
| P15_005 | 105,447,580 | 92,375,438 | 87.6 | 65,277,362 | 61.9 | 55.5 | 6,733,466 | 6.4 |
| P15_057 | 99,844,384 | 95,111,640 | 95.3 | 82,464,204 | 82.6 | 43.7 | 38,784,022 | 38.8 |
| P15_065 | 102,050,394 | 94,971,810 | 93.1 | 82,445,942 | 80.8 | 39.1 | 42,561,264 | 41.7 |
| P15_073 | 107,014,782 | 101,544,652 | 94.9 | 90,444,746 | 84.5 | 46.2 | 40,981,674 | 38.3 |
| P15_020 | 93,820,826 | 88,944,596 | 94.8 | 78,847,480 | 84.0 | 62.4 | 20,311,968 | 21.6 |
| P15_018 | 97,014,038 | 89,173,708 | 91.9 | 75,971,426 | 78.3 | 24.9 | 51,785,968 | 53.4 |
| P15_004 | 86,078,536 | 76,293,050 | 88.6 | 60,122,444 | 69.8 | 28.1 | 35,887,018 | 41.7 |
| P15_041 | 110,028,916 | 103,049,878 | 93.7 | 88,982,444 | 80.9 | 38.7 | 46,359,792 | 42.1 |
