# Supplementary material for: Combining explainable machine learning, demographic and multi-omic data to inform precision medicine strategies for inflammatory bowel disease
Source: PLoS One. 2022 Feb 23;17(2):e0263248. doi: 10.1371/journal.pone.0263248 (PMC8865677; doi:10.1371/journal.pone.0263248)
Supplement: S4 Table — (DOCX) [file pone.0263248.s008.docx]

**Table S4. Hyper-parameters of the best ML models selected by Hyper-parameter Optimization**

| Drug | Feature set | Best Model | Hyper-parameters |
| --- | --- | --- | --- |
| BIRB796 10nM | Demographic + correlated meds | KNN | KNeighborsRegressor(algorithm='auto', leaf_size=30, metric='euclidean',  metric_params=None, n_jobs=None, n_neighbors=4, p=2,  weights='uniform') |
| BIRB796 10nM | Demographic + correlated meds + selected SNPs | KNN | KNeighborsRegressor(algorithm='auto', leaf_size=30, metric='manhattan',  metric_params=None, n_jobs=None, n_neighbors=7, p=2,  weights='uniform') |
| BIRB796 100nM | Demographic + correlated meds (per drug) + known SNPs only | RF | RandomForestRegressor(bootstrap=True, ccp_alpha=0.0, criterion='mse',  max_depth=33, max_features='auto', max_leaf_nodes=None,  max_samples=None, min_impurity_decrease=0.0,  min_impurity_split=None, min_samples_leaf=4,  min_samples_split=5, min_weight_fraction_leaf=0.0,  n_estimators=72, n_jobs=None, oob_score=False,  random_state=42, verbose=0, warm_start=False) |
| SEBPRED 1uM | Demographic + correlated meds (per drug) + known SNPs only | KNN | KNeighborsRegressor(algorithm='auto', leaf_size=30, metric='manhattan',  metric_params=None, n_jobs=None, n_neighbors=4, p=2,  weights='uniform') |
| SEBPRED 100nM | Demographic + correlated meds (per drug) + known SNPs only | RF | RandomForestRegressor(bootstrap=True, ccp_alpha=0.0, criterion='mse',  max_depth=63, max_features='sqrt', max_leaf_nodes=None,  max_samples=None, min_impurity_decrease=0.0,  min_impurity_split=None, min_samples_leaf=4,  min_samples_split=10, min_weight_fraction_leaf=0.0,  n_estimators=40, n_jobs=None, oob_score=False,  random_state=42, verbose=0, warm_start=False) |
| 5ASA | Demographic + correlated meds (per drug) + known SNPs only | RF | RandomForestRegressor(bootstrap=True, ccp_alpha=0.0, criterion='mse',  max_depth=40, max_features='sqrt', max_leaf_nodes=None,  max_samples=None, min_impurity_decrease=0.0,  min_impurity_split=None, min_samples_leaf=2,  min_samples_split=10, min_weight_fraction_leaf=0.0,  n_estimators=26, n_jobs=None, oob_score=False,  random_state=42, verbose=0, warm_start=False) |
